# Sesquiterpenes from the soil-derived fungus *Trichoderma citrinoviride* PSU-SPSF346

**DOI:** 10.3762/bjoc.18.50

**Published:** 2022-04-29

**Authors:** Wiriya Yaosanit, Vatcharin Rukachaisirikul, Souwalak Phongpaichit, Sita Preedanon, Jariya Sakayaroj

**Affiliations:** 1 Division of Physical Science and Center of Excellence for Innovation in Chemistry, Faculty of Science, Prince of Songkla University, Hat Yai, Songkhla 90110, Thailand; 2 Division of Biological Science, Faculty of Science, Prince of Songkla University, Hat Yai, Songkhla 90110, Thailand; 3 National Biobank of Thailand (NBT), National Science and Technology for Development Agency (NSTDA), Thailand Science Park, Klong Luang, Pathumthani 12120, Thailand; 4 School of Science, Walailak University, Thasala, Nakhonsithammarat 80160, Thailand

**Keywords:** antimicrobial activity, cytotoxic activity, γ-lactone, soil-derived fungus, sesquiterpene, *Trichoderma citrinoviride*

## Abstract

Two new sesquiterpenes, trichocitrinovirenes A (**1**) and B (**2**), and five known compounds including four structurally related sesquiterpenes and one γ-lactone were isolated from the soil-derived fungus *Trichoderma citrinoviride* PSU-SPSF346. The structures were identified by analysis of their spectroscopic data. The relative configuration was assigned based on NOEDIFF data. The absolute configuration of compound **1** was established according to specific rotations and ECD data while that of compound **2** was proposed based on biosynthetic considerations. Compound **2** possesses a rare bicyclic sesquiterpene skeleton. The antimicrobial and cytotoxic activities of the isolated compounds were evaluated.

## Introduction

The fungus *Trichoderma citrinoviride* produces structurally diverse secondary metabolites including diterpenes [1–2], alkaloids [3], sorbicillinoids [4–5], long chain alcohols [6], and cyclonerane sesquiterpenes [7]. Some of them display antibacterial [1–2,7], cytotoxic [3], anti-inflammatory [4], and antimicroalgal [7] activities. Based on these data, the investigation of secondary metabolites from this fungus is still limited. In our ongoing search for antimicrobial secondary metabolites from soil-derived fungi, *T*. *citrinoviride* PSU-SPSF346 was isolated from a soil sample collected from the Sirindhorn Peat Swamp Forest, Narathiwat Province, Thailand. The crude mycelial extract of *T*. *citrinoviride* PSU-SPSF346 displayed antimicrobial activities against *Staphylococcus aureus*, methicillin-resistant *S*. *aureus*, and *Cryptococcus neoformans* ATCC90113 with MIC values of 128, 200 and 64 μg/mL, respectively. Herein, we report the isolation and structure elucidation as well as antimicrobial and cytotoxic activities of some isolated compounds which were obtained in sufficient amount.

## Results and Discussion

**Figure 1:**
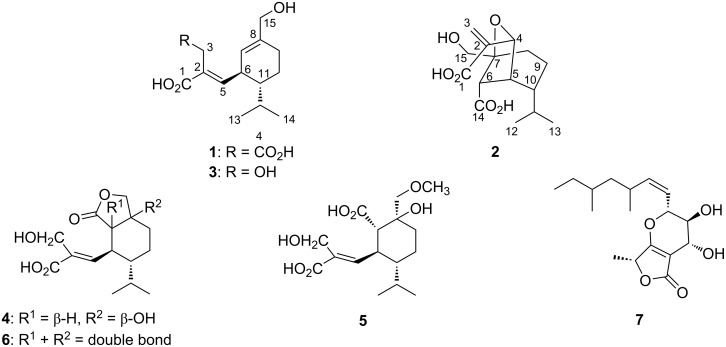
Structures of compounds **1**–**7** isolated from *Trichoderma citrinoviride* PSU-SPSF346.

Chemical investigation of the broth and mycelial extracts of *T*. *citrinoviride* PSU-SPSF346 by various chromatography techniques led to the isolation of two new sesquiterpenes, trichocitrinovirenes A (**1**) and B (**2**), four known structurally related sesquiterpenes including gliocladic acid (**3**), hydroheptelidic acid (**4**), and xylaric acids B (**5**) and D (**6**) [8], as well as one known γ-lactone, fusidilactone A (**7**) [9] (Figure 1). Their structures were elucidated on the basis of analysis of their spectroscopic data including IR, UV, NMR, and MS. The relative configuration was assigned based on NOEDIFF data. Furthermore, the absolute configuration of compound **1** was determined by comparison of its specific rotation and electronic circular dichroism (ECD) data with those of compound **3**, whereas that of compound **2** was proposed based on biosynthetic considerations. The structures of the known compounds were further confirmed by comparison of their ^1^H and ^13^C NMR spectroscopic data, specific rotations, and ECD data with those previously reported.

**Figure 2:**
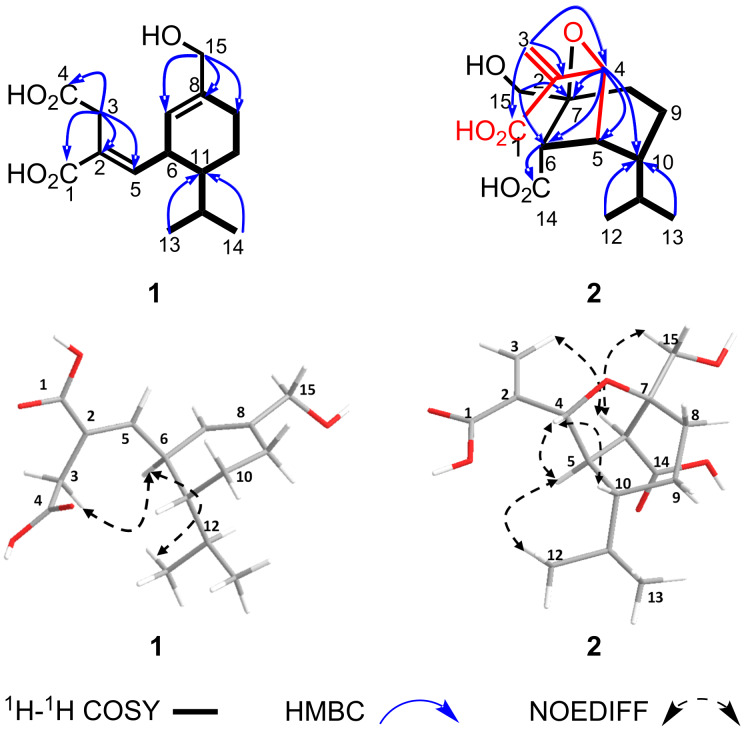
^1^H-^1^H COSY, key HMBC, and NOEDIFF data of compounds **1** and **2**.

**Figure 3:**
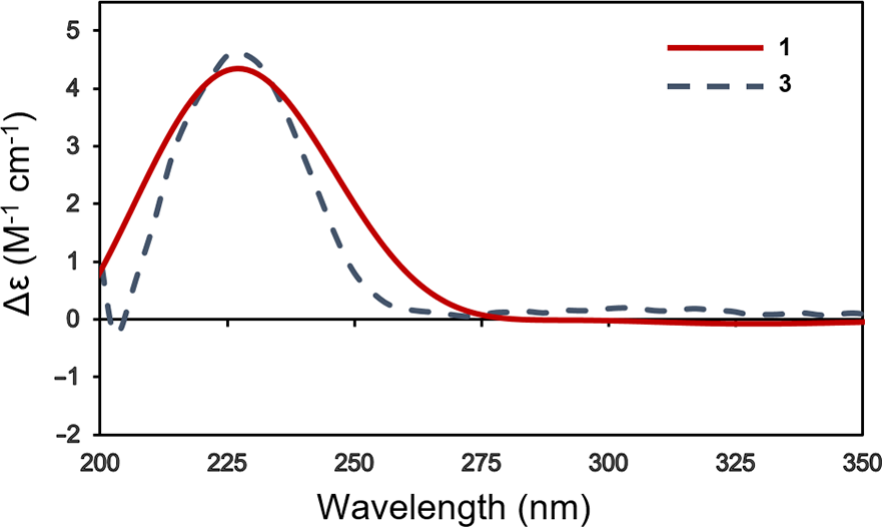
ECD spectra of compounds **1** and **3** in MeOH.

Trichocitrinovirene A (**1**) was isolated as a colorless gum and had the molecular formula C_15_H_22_O_5_ on the basis of the HRESIMS peak at *m*/*z* 305.1359 [M + Na]^+^. The IR spectrum exhibited absorption bands at 3336, 1684, and 1649 cm^−1^ for hydroxy, conjugated carboxyl carbonyl, and double bond functional groups, respectively [10]. The ^1^H NMR spectrum (Table 1) displayed characteristic signals for two olefinic protons of two trisubstituted alkenes (δ_H_ 6.64, d, *J* = 10.5 Hz, and 5.31, s, each 1H), two methine protons (δ_H_ 3.06 and 1.35, each m, 1H), one set of equivalent oxymethylene protons (δ_H_ 3.92, s, 2H), three sets of nonequivalent methylene protons (δ_H_ 3.40 and 3.30, each d, *J* = 16.8 Hz, 1H; δ_H_ 2.13 and 2.04, each m, 1H; δ_H_ 1.81 and 1.39, each m, 1H), and an isopropyl group (δ_H_ 1.71, m, 1H, and δ_H_ 0.95 and 0.82, each d, *J* = 6.9 Hz, 3H). The ^13^C NMR spectrum (Table 1) consisted of signals for two carboxyl carbonyl carbons (δ_C_ 175.1 and 171.1), two olefinic quaternary carbons (δ_C_ 141.1 and 128.1), two olefinic methine carbons (δ_C_ 149.7 and 123.5) and three methine carbons (δ_C_ 46.8, 40.7 and 29.8), one oxymethylene carbon (δ_C_ 67.0), three methylene carbons (δ_C_ 33.6, 26.7 and 22.4), and two methyl carbons (δ_C_ 21.7 and 17.3). These NMR spectroscopic data were similar to those of compound **3** except for the replacement of signals for the nonequivalent oxymethylene protons (δ_H_ 4.37 and 4.32, H_ab_-3; δ_C_ 57.4, C-3) in compound **3** with signals of the nonequivalent methylene protons resonating at higher field (δ_H_ 3.40 and 3.30, each d, *J* = 16.8 Hz, 1H; δ_C_ 33.6, C-3), and an additional signal for a carboxyl carbonyl carbon (δ_C_ 175.1) in compound **1**. The HMBC cross peaks of these nonequivalent methylene protons with C-1 (δ_C_ 171.1), C-2 (δ_C_ 128.1), C-4 (δ_C_ 175.1), and C-5 (δ_C_ 149.7) (Figure 2) together with the chemical shift of C-3 indicated that the 3-OH group in compound **3** was replaced by a carboxyl group in compound **1**. The relative configuration was determined by the NOEDIFF data (Figure 2). A signal enhancement of H-7, but not H-5, after irradiation of H_ab_-3, indicated an *E*-configuration of the trisubstituted α,β-unsaturated carboxylic acid. In addition, a *trans* relationship between H-6 and H-11 was established according to signal enhancement of H-12 (δ_H_ 1.71), H_3_-13, and H_3_-14 after irradiation of H-6. The absolute configuration at C-6 was assigned as *R* based on the experimental ECD spectrum of compound **1** which showed a positive Cotton effect at 227 nm (Δε +4.3, *c* 0.0008, MeOH), the same sign as that of compound **3**, (λ_max_: 227 nm, Δε +4.8, *c* 0.0008 M, MeOH) [8] (Figure 3). Accordingly, C-11 had an *R* configuration. The observed specific rotation of compound **1**, 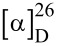 +46.1 (*c* 0.67, MeOH), was similar to that of compound **3**, 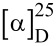 +41.4 (*c* 0.67, MeOH) [8], thus supporting the assigned absolute configurations at both C-6 and C-11 of compound **1**. Therefore, **1** was a 3-carboxyl derivative of compound **3**.

**Table 1:** The NMR data of compounds **1** and **2** in CD_3_OD.

No.	**1**			**2**	
			
	δ_C_, C-type	δ_H_, mult. (*J* [Hz])		δ_C_, C-type	δ_H_, mult. (*J* [Hz])

1	171.1, C			174.5, C	
2	128.1, C			150.2, C	
3	33.6, CH_2_	a: 3.40, d (16.8)		118.7, CH_2_	a: 5.93, s
		b: 3.30, d (16.8)			b: 5.52, s
4	175.1, C			84.6, CH	4.72, s
5	149.7, CH	6.64, d (10.5)		47.0, CH	2.81, brs
6	40.7, CH	3.06, m		51.4, CH	2.63, d (3.0)
7	123.5, CH	5.31, s		83.4, C	
8	141.1, C			29.3, CH_2_	a: 2.45, ddd (14.5, 9.5, 6.5) b: 1.37, m
9	26.7, CH_2_	a: 2.13, m b: 2.04, m		22.5, CH_2_	a: 1.84, m b: 1.64, m
10	22.4, CH_2_	a: 1.81, m b: 1.39, m		49.0, CH	1.35, m
11	46.8, CH	1.35, m		30.6, CH	1.83, m
12	29.8, CH	1.71, m		23.0, CH_3_	1.06, d (6.5)
13	17.3, CH_3_	0.82, d (6.9)		22.5, CH_3_	0.88, d (6.5)
14	21.7, CH_3_	0.95, d (6.9)		179.6, C	
15	67.0, CH_2_	3.92, s		69.2, CH_2_	a: 3.69, d (10.5) b: 3.65, d (10.5)

**Figure 4:**
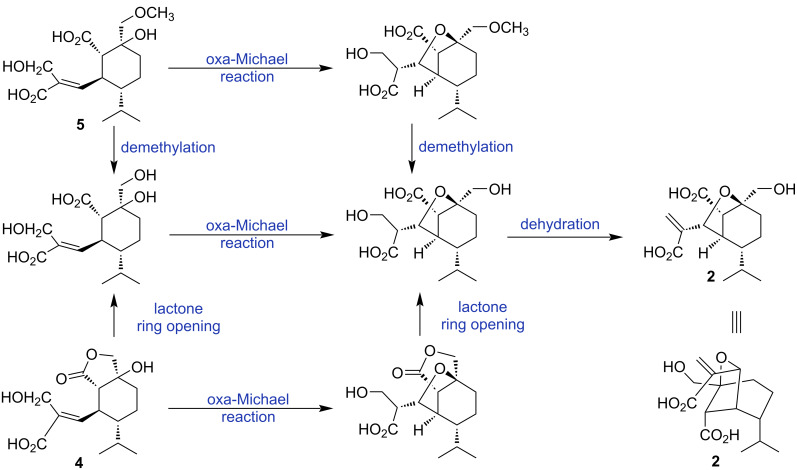
Proposed biosynthetic pathway for compound **2**.

Trichocitrinovirene B (**2**) was isolated as a colorless gum. The molecular formula C_15_H_22_O_6_ was determined on the basis of the HRESIMS peak at *m*/*z* 321.1320 [M + Na]^+^. The IR spectrum was similar to that of compound **1**, indicating that they possessed similar functional groups. The ^1^H NMR spectrum (Table 1) displayed characteristic signals for two geminal olefinic protons (δ_H_ 5.93 and 5.52, each s, 1H), one oxymethine proton (δ_H_ 4.72, s, 1H), three methine protons (δ_H_ 2.81, brs, 1H, 2.63, d, *J* = 3.0 Hz, 1H and 1.35, m, 1H), one set of nonequivalent oxymethylene protons (δ_H_ 3.69 and 3.65, each d, *J* = 10.5 Hz, 1H), two sets of nonequivalent methylene protons (δ_H_ 2.45, ddd, *J* = 14.5, 9.5 and 6.5 Hz, 1H and 1.37, m, 1H; δ_H_ 1.84 and 1.64, each m, 1H), and an isopropyl group (δ_H_ 1.83, m, 1H, and δ_H_ 1.06 and 0.88, each d, *J* = 6.5 Hz, 3H). The ^13^C NMR spectrum (Table 1) consisted of signals for two carboxyl carbonyl carbons (δ_C_ 179.6 and 174.5), two quaternary carbons (one olefinic carbon, δ_C_ 150.2, and one oxycarbon, δ_C_ 83.4), one oxymethine carbon (δ_C_ 84.6), four methine carbons (δ_C_ 51.4, 49.0, 47.0 and 30.6), one oxymethylene carbon (δ_C_ 69.2), three methylene carbons (δ_C_ 118.7, 29.3 and 22.5), and two methyl carbons (δ_C_ 23.0 and 22.5). The ^1^H-^1^H COSY correlations (Figure 2) of H-6 (δ_H_ 2.63)/H-5 (δ_H_ 2.81), H-5/H-10 (δ_H_ 1.35), and H_ab_-9 (δ_H_ 1.84 and 1.64)/H_ab_-8 (δ_H_ 2.45 and 1.37) and H-10, and the HMBC correlations (Figure 2) from H_3_-12 (δ_H_ 1.06) and H_3_-13 (δ_H_ 0.88) of the isopropyl group to C-10 (δ_C_ 49.0) and H_ab_-15 (δ_H_ 3.69 and 3.65) to C-6 (δ_C_ 51.4), C-7 (δ_C_ 83.4), and C-8 (δ_C_ 29.3) as well as the chemical shifts of C-7 and C-15 (δ_C_ 69.2) constructed a cyclohexane ring with both a hydroxymethyl group and an oxy substituent at C-7, the isopropyl group at C-10 and other substituents at C-5 (δ_C_ 47.0) and C-6. The substituent at C-6 was assigned as a carboxyl group on the basis of the HMBC correlation of H-6 with the carboxyl carbonyl carbon (C-14, δ_C_ 179.6). The HMBC correlations of the geminal olefinic protons (H_ab_-3, δ_H_ 5.93 and 5.52) to the remaining carboxyl carbonyl carbon (C-1, δ_C_ 174.5), C-2 (δ_C_ 150.2) and C-4 (δ_C_ 84.6) and H-4 (δ_H_ 4.72) to C-2 and C-3 (δ_C_ 118.7) and the chemical shift of C-4 constructed a propenoic acid unit with a 1,1-disubstituted oxymethyl substituent at C-2. The HMBC correlations of H-4 of this unit with C-5, C-6, C-7, and C-10 as well as the chemical shifts of C-4 and C-7 established an ether bond between C-4 and C-7 and a C–C bond between C-4 and C-5 to form a bicyclic skeleton. The relative configuration was determined by the NOEDIFF data (Figure 2). The signal enhancement of H-5 and H-10 upon irradiation of H-4 indicated their close proximity and the orientation of the isopropyl group at an α-position. Irradiation of H-6 enhanced the signal intensities of H_b_-3, H-5, and H_ab_-15, indicating that the carboxyl moiety was α-orientated. Biosynthetically, compound **2** might be derived from compound **4** or compound **5** by oxa-Michael reaction of 7-OH to the α,β-unsaturated carboxylic acid moiety to form a tetrahydrofuran unit followed by ring opening of the lactone moiety and demethylation, respectively (Figure 4). Subsequent dehydration would afford compound **2** with an α,β*-*unsaturated carboxylic acid moiety. Alternatively, the ring opening of compound **4** and demethylation of compound **5** would occur prior to the oxa-Michael reaction. Accordingly, the absolute configurations at C-5, C-6, C-7, and C-10 of compound **2** were proposed to be 5*S*, 6*S*, 7*S*, and 10*R* identical to those of the co-metabolites **4** and **5**. The absolute configuration at C-4 was thus assigned to be *R*. Therefore, compound **2** is a rare bicyclic sesquiterpene.

The isolated compounds **1** and **3**–**6** with sufficient amount were evaluated for their antibacterial activity against *S*. *aureus* ATCC25923 and methicillin-resistant *S*. *aureus* SK1, antifungal activity against *C*. *neoformans* ATCC90113 as well as cytotoxic activity against KB, MCF-7, and noncancerous Vero (African green monkey kidney fibroblast) cells. None of them showed antimicrobial activity against the tested pathogenic microorganisms at the concentration of 200 μg/mL. These results indicate either a synergistic effect and/or that the active components were not isolated. In addition, these compounds were inactive against the tested cell lines at the concentration of 50 μg/mL.

## Conclusion

The investigation of the crude extracts of the soil-derived fungus *T*. *citrinoviride* PSU-SPSF346 resulted in the isolation of seven compounds including two new (**1** and **2**) and four known sesquiterpenes (**3**–**6**), and one known γ-lactone (**7**). Sesquiterpenes of this type were previously isolated from *T*. *virens* [11–12], *T*. *reesei* [10], and *Xylaria* sp. [8,13] whereas compound **7** was obtained from *Fusidium* sp. [9] and *T*. *hypoxylon* [14]. Therefore, this is the first report on the isolation of these types of compounds from the fungus *T*. *citrinoviride*. In addition, compound **2** is a rare bicyclic sesquiterpene. Unfortunately, none of the tested compounds **1** and **3**–**6** displayed antimicrobial and cytotoxic activities.

## Experimental

### General experimental procedures

Infrared (IR) spectra were recorded with a Perkin-Elmer spectrum BX FTIR spectrometer. Ultraviolet (UV) spectra were obtained using a Shimadzu UV-2600 UV–vis spectrophotometer in MeOH. ECD spectra were recorded on a JASCO J-815 polarimeter. ^1^H and ^13^C nuclear magnetic resonance (^1^H and ^13^C NMR) spectra were recorded on 300 and 500 MHz Bruker FTNMR Ultra Shield^TM^ spectrometers. Specific rotations were measured with a JASCO P-2000 polarimeter. ESI-TOF mass spectra were obtained using a TOF/Q-TOF Mass spectrometer. Thin-layer chromatography (TLC) and preparative TLC were performed on silica gel 60 GF_254_ (Merck). Column chromatography (CC) was conducted on silica gel (Merck) type 100 (70–230 mesh ASTM) and type 60 (230–400 mesh ASTM), Sephadex LH-20, or reversed-phase C_18_ silica gel.

### Fungal material

The fungus PSU-SPSF346 was isolated from a soil sample collected from the Sirindhorn Peat Swamp Forest, Narathiwat Province, Thailand. This fungus was deposited as BCC88125 at BIOTEC Culture Collection (BCC), National Center for Genetic Engineering and Biotechnology (BIOTEC), Thailand. The fungus SPSF346 was identified based on its morphological and molecular characteristics. The molecular analysis of the internal transcribed spacers (ITS) (GenBank accession number MH997885) and partial large subunit (LSU) (GenBank accession number MH997897) ribosomal RNA gene revealed that the fungus PSU-SPSF346 had close relationships with several strains of *Trichoderma citrinoviride* with 99% nucleotide identity for both DNA regions. Therefore, this fungus can be identified as *Trichoderma citrinoviride*.

### Fermentation, extraction, and isolation

The crude broth ethyl acetate (BE, 16.5 g) and the mycelial ethyl acetate (CE, 2.6 g) extracts were obtained as a dark brown gum and yellow-brown gum, respectively, using the same procedure as previously described [15]. The broth extract was separated by CC over Sephadex LH-20 using a mixture of MeOH/CH_2_Cl_2_ 1:1 to afford five fractions (A1–A5). Fraction A4 (7.0 g) was purified by CC over Sephadex LH-20 using a mixture of MeOH/CH_2_Cl_2_ 1:3 to obtain seven subfractions (A4A–A4G). Subfraction A4F (3.0 g) was purified using the same procedure as fraction A4 to afford eight subfractions (A4F1–A4F8). Subfraction A4F6 (770.3 mg) was separated by CC over silica gel using a mixture of EtOAc/CH_2_Cl_2_/MeOH 18:1:1 to give 12 subfractions (A4F6A–A4F6L). Subfraction A4F6H contained compound **3** (91.3 mg). Subfraction A4F6J (532.4 mg) afforded compound **4** (178.3 mg) after purification by CC over reversed-phase C_18_ silica gel using a mixture of MeOH/H_2_O 1:1. Subfraction A4F6K (30.1 mg) was purified using the same procedure as subfraction A4F6J followed by CC over Sephadex LH-20 using a mixture of MeOH/CH_2_Cl_2_ 3:1 to afford compound **1** (4.1 mg). Subfraction A4G (80.0 mg) was subjected to CC over reversed-phase C_18_ silica gel using a mixture of MeOH/H_2_O 3:2 to yield six subfractions (A4G1–A4G6). Subfraction A4G2 (18.2 mg) was separated by CC over silica gel using a mixture of CH_2_Cl_2_/hexane/MeOH 17:2:1 followed by CC over Sephadex LH-20 using MeOH to obtain compound **2** (2.6 mg). The mycelial EtOAc extract (2.6 g) was fractionated by CC over Sephadex LH-20 using a mixture of MeOH/CH_2_Cl_2_ 1:1 to give five fractions (B1–B5). Fraction B3 (892.5 mg) was purified by CC over silica gel using a mixture of MeOH/CH_2_Cl_2_ 3:97 to give ten subfractions (B3A–B3J). Subfraction B3E (81.5 mg) was separated by CC over silica gel using a mixture of acetone/hexane 1:4 to afford five subfractions (B3E1–B3E5). Subfraction B3E2 (5.1 mg) was purified using the same procedure as fraction A4 to afford compound **7** (2.8 mg). Subfraction B3I (454.1 mg) was separated using the same procedure as subfraction A4F6J to provide seven subfractions (B3I1–B3I7). Subfraction B3I3 (39.1 mg) was purified by CC over reversed-phase C_18_ silica gel using a mixture of MeOH/H_2_O 2:3 to give compound **6** (5.3 mg). Subfraction B3J (89.9 mg) was further purified using the same procedure as subfraction A4F6J to give seven subfractions (B3J1–B3J7). Subfraction B3J3 (6.0 mg) was then washed with acetone to afford compound **5** (3.7 mg).

**Trichocitrinovirene A (1):** Colorless gum; 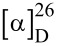 +46.1 (*c* 0.67, MeOH); UV (MeOH) λ_max_, nm (log ε): 210 (3.32); ECD (MeOH, *c* 0.0008) λ_max_, nm (Δε): 227 (+4.3); IR (neat) ν_max_: 3336, 1684, 1649 cm^−1^; ^1^H and ^13^C NMR (CD_3_OD) see Table 1; HRMS–ESI (*m*/*z*): [M + Na]^+^ calcd for C_15_H_22_O_5_Na, 305.1356; found, 305.1359.

**Trichocitrinovirene B (2):** Colorless gum; 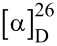 +44.6 (*c* 0.67, MeOH); UV (MeOH) λ_max_, nm (log ε): 210 (3.67); IR (neat) ν_max_: 3386, 1683, 1645 cm^−1^; ^1^H and ^13^C NMR (CD_3_OD) see Table 1; HRMS–ESI (*m*/*z*): [M + Na]^+^ calcd for C_15_H_22_O_6_Na, 321.1309; found, 321.1320.

### Antimicrobial assay

Antimicrobial activity was evaluated according to the Clinical and Laboratory Standards Institute [16]. Vancomycin was used as a positive control for *S*. *aureus* and methicillin-resistant *S*. *aureus* and exhibited MIC values of 0.25 and 1.0 μg/mL, respectively. Amphotericin B was used as a positive control for *C*. *neoformans* ATCC90113 and displayed a MIC value of 0.25 μg/mL.

### Cytotoxicity assay

The activity assay against African green monkey kidney fibroblast (Vero) cells was performed in triplicate employing the method described by Hunt and co-workers [17]. Ellipticine, the standard drug, displayed an IC_50_ value of 4.06 μM. The activities against KB and MCF-7 cell lines were evaluated using the resazurin microplate assay [18]. Doxorubicin was used as a standard drug for KB and MCF-7 cell lines and displayed IC_50_ values of 1.21 and 15.84 μM, respectively.

## Supporting Information

File 1 HRESIMS profiles for compounds **1** and **2** and copies of NMR spectra for compounds **1**–**7**.
