# Supplementary material for: Sesquiterpenes from the soil-derived fungus Trichoderma citrinoviride PSU-SPSF346
Source: Beilstein J Org Chem. 2022 Apr 29;18:479–85. doi: 10.3762/bjoc.18.50 (PMC9062651; doi:10.3762/bjoc.18.50)
Supplement: File 1 — HRESIMS profiles for compounds 1 and 2 and copies of NMR spectra for compounds 1–7. [file Beilstein_J_Org_Chem-18-479-s001.pdf]

BEILSTEIN JOURNAL OF ORGANIC CHEMISTRY

Supporting Information

for

Sesquiterpenes from the soil-derived fungus *Trichoderma citrinoviride* PSU-SPSF346

Wiriya Yaosanit, Vatcharin Rukachaisirikul, Souwalak Phongpaichit, Sita Preedanon and Jariya Sakayaroj

Beilstein J. Org. Chem. **2022,** *18,* 479–485. doi:10.3762/bjoc.18.50

HRESIMS profiles for compounds 1 and 2 and copies of NMR spectra for compounds 1–7

License and Terms: This is a supporting information file under the terms of the Creative Commons Attribution License (https://creativecommons.org/licenses/by/4.0). Please note that the reuse, redistribution and reproduction in particular requires that the author(s) and source are credited and that individual graphics may be subject to special legal provisions.
The license is subject to the *Beilstein Journal of Organic Chemistry* terms and conditions: (https://www.beilstein-journals.org/bjoc/terms)

Table of contents

		Page
**Figure S1**	^{1}H NMR spectrum (300 MHz, CD_3OD) of compound **1**	S2
**Figure S2**	^{13}C NMR spectrum (75 MHz, CD_3OD) of compound **1**	S2
**Figure S3**	HMQC spectrum (CD_3OD) of compound **1**	S3
**Figure S4**	HMBC spectrum (CD_3OD) of compound **1**	S4
**Figure S5**	^{1}H-^{1}H COSY spectrum (CD_3OD) of compound **1**	S5
**Figure S6**	HRESI mass spectrum of compound **1**	S6
**Figure S7**	^{1}H NMR spectrum (500 MHz, CD_3OD) of compound **2**	S7
**Figure S8**	^{13}C NMR spectrum (125 MHz, CD_3OD) of compound **2**	S7
**Figure S9**	HMQC spectrum (CD_3OD) of compound **2**	S8
**Figure S10**	HMBC spectrum (CD_3OD) of compound **2**	S9
**Figure S11**	^{1}H-^{1}H COSY spectrum (CD_3OD) of compound **2**	S10
**Figure S12**	HRESI mass spectrum of compound **2**	S11
**Figure S13**	^{1}H NMR spectrum (300 MHz, CD_3OD) of compound **3**	S12
**Figure S14**	^{13}C NMR spectrum (75 MHz, CD_3OD) of compound **3**	S12
**Figure S15**	^{1}H NMR spectrum (300 MHz, acetone-d_6) of compound **4**	S13
**Figure S16**	^{13}C NMR spectrum (75 MHz, acetone-d_6) of compound **4**	S13
**Figure S17**	^{1}H NMR spectrum (300 MHz, CD_3OD) of compound **5**	S14
**Figure S18**	^{13}C NMR spectrum (75 MHz, CD_3OD) of compound **5**	S14
**Figure S19**	^{1}H NMR spectrum (300 MHz, CD_3OD) of compound **6**	S15
**Figure S20**	^{13}C NMR spectrum (75 MHz, CD_3OD) of compound **6**	S15
**Figure S21**	^{1}H NMR spectrum (500 MHz, $CDCl_3$) of compound **7**	S16
**Figure S22**	^{13}C NMR spectrum (125 MHz, $CDCl_3$) of compound **7**	S16
	References	S17

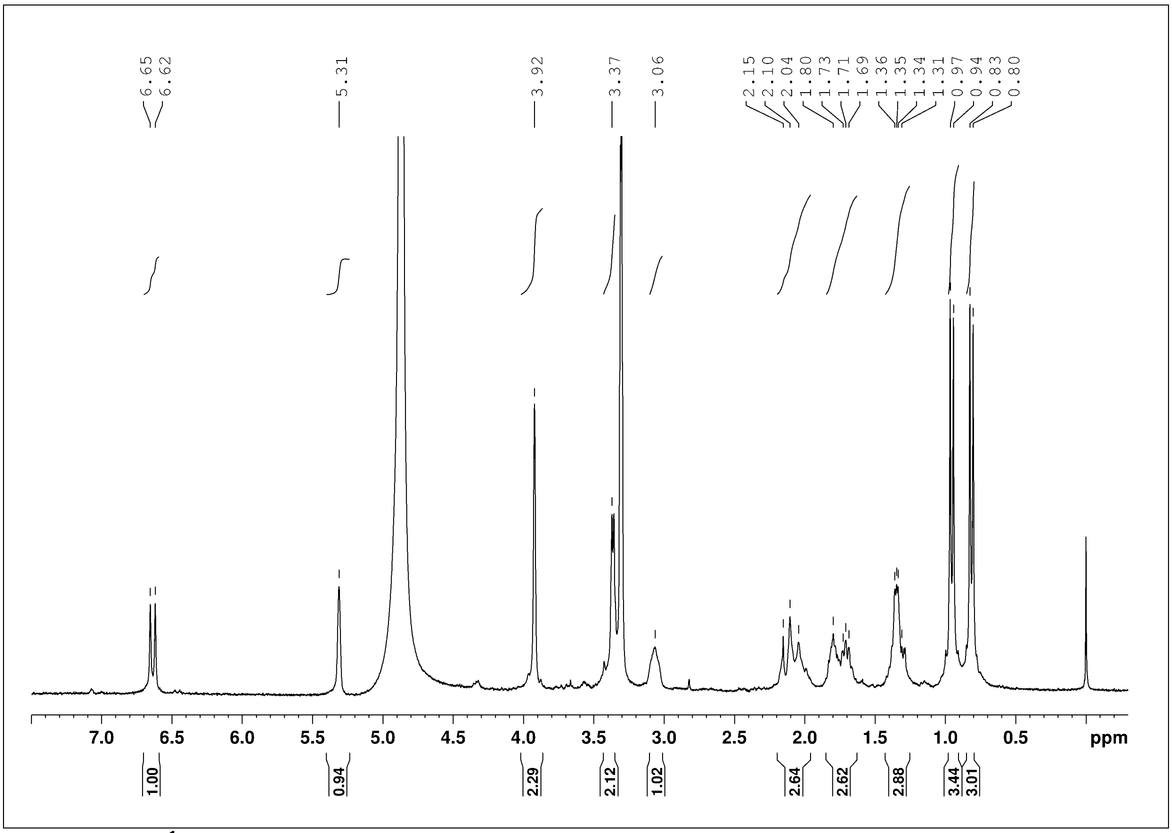

Figure S1. ^{1}H NMR spectrum (300 MHz, CD_3OD) of compound **1**

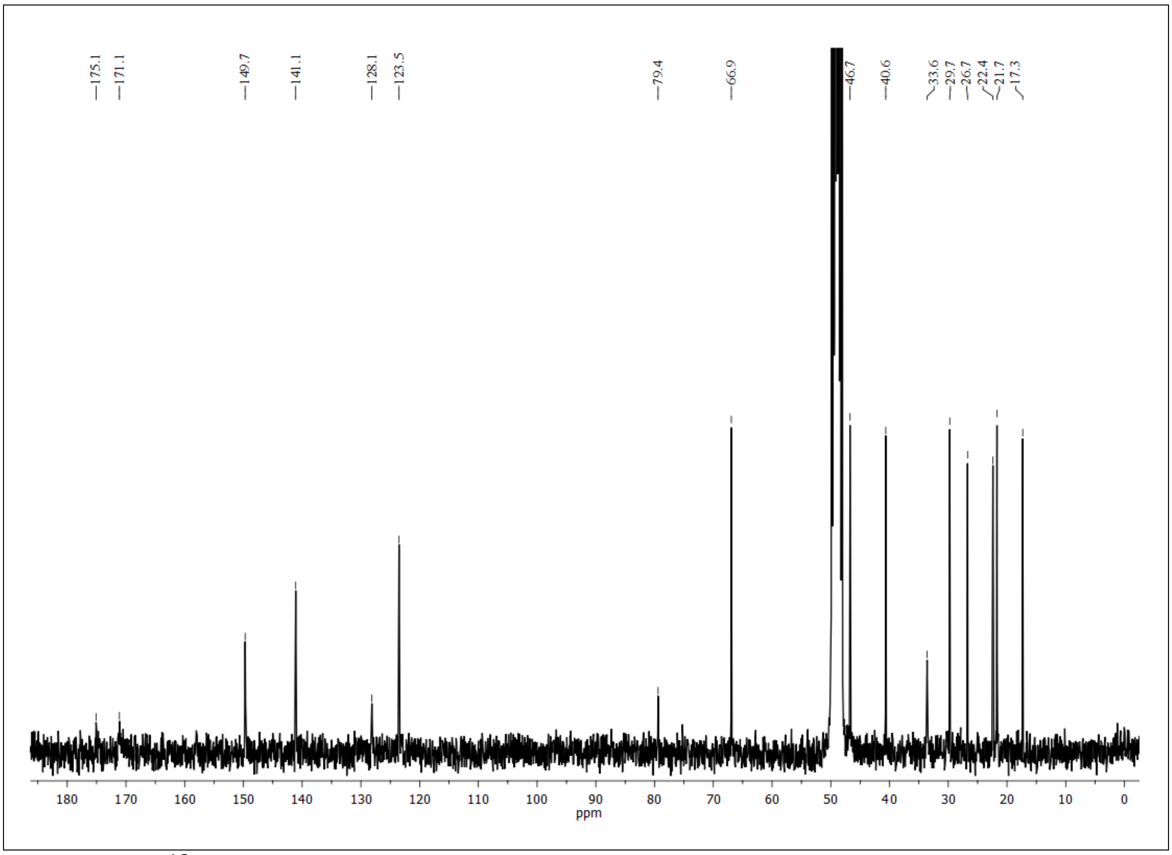

Figure S2. ^{13}C NMR spectrum (75 MHz, CD_3OD) of compound **1**

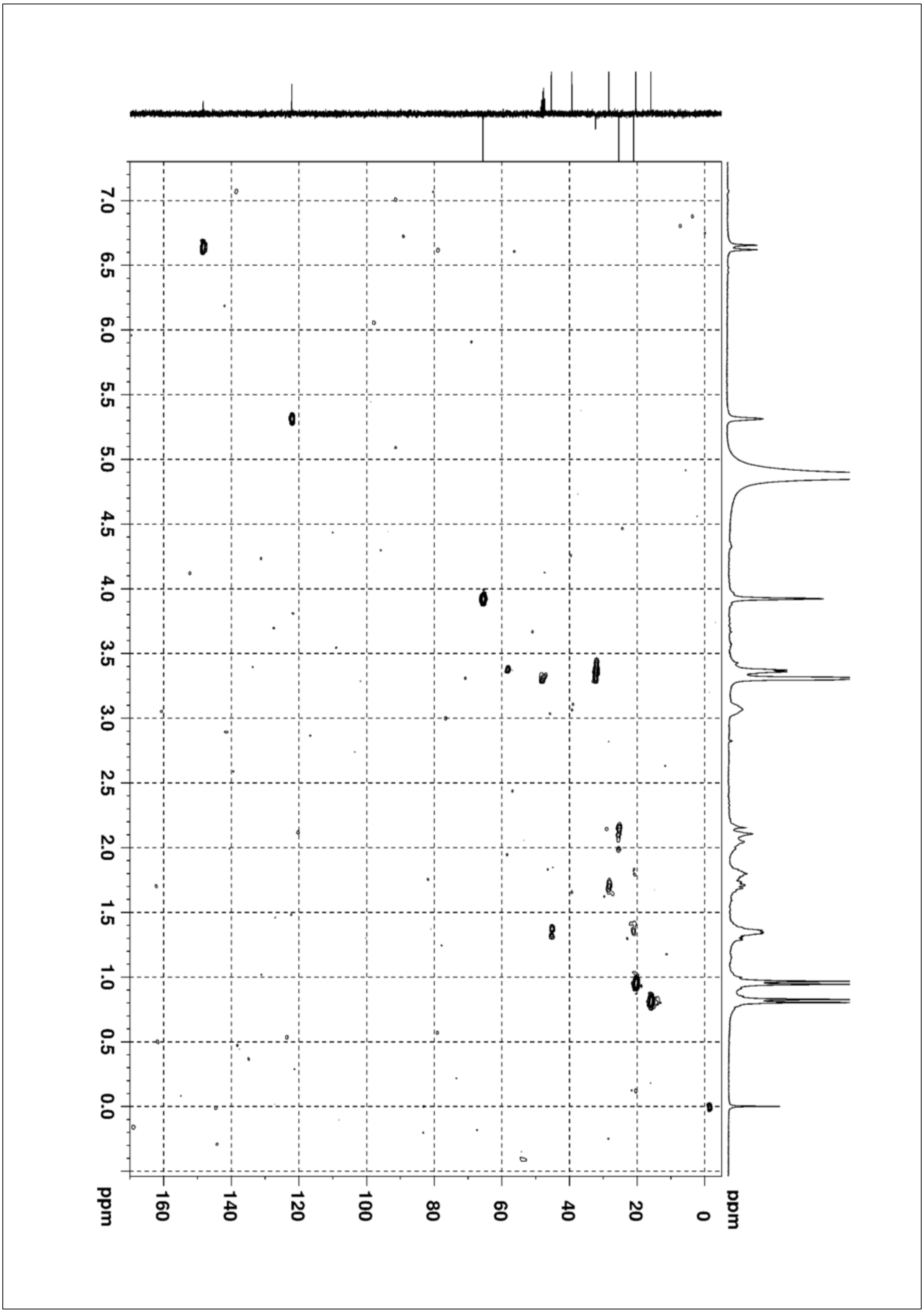

Figure S3. HMQC spectrum (CD_3OD) of compound **1**

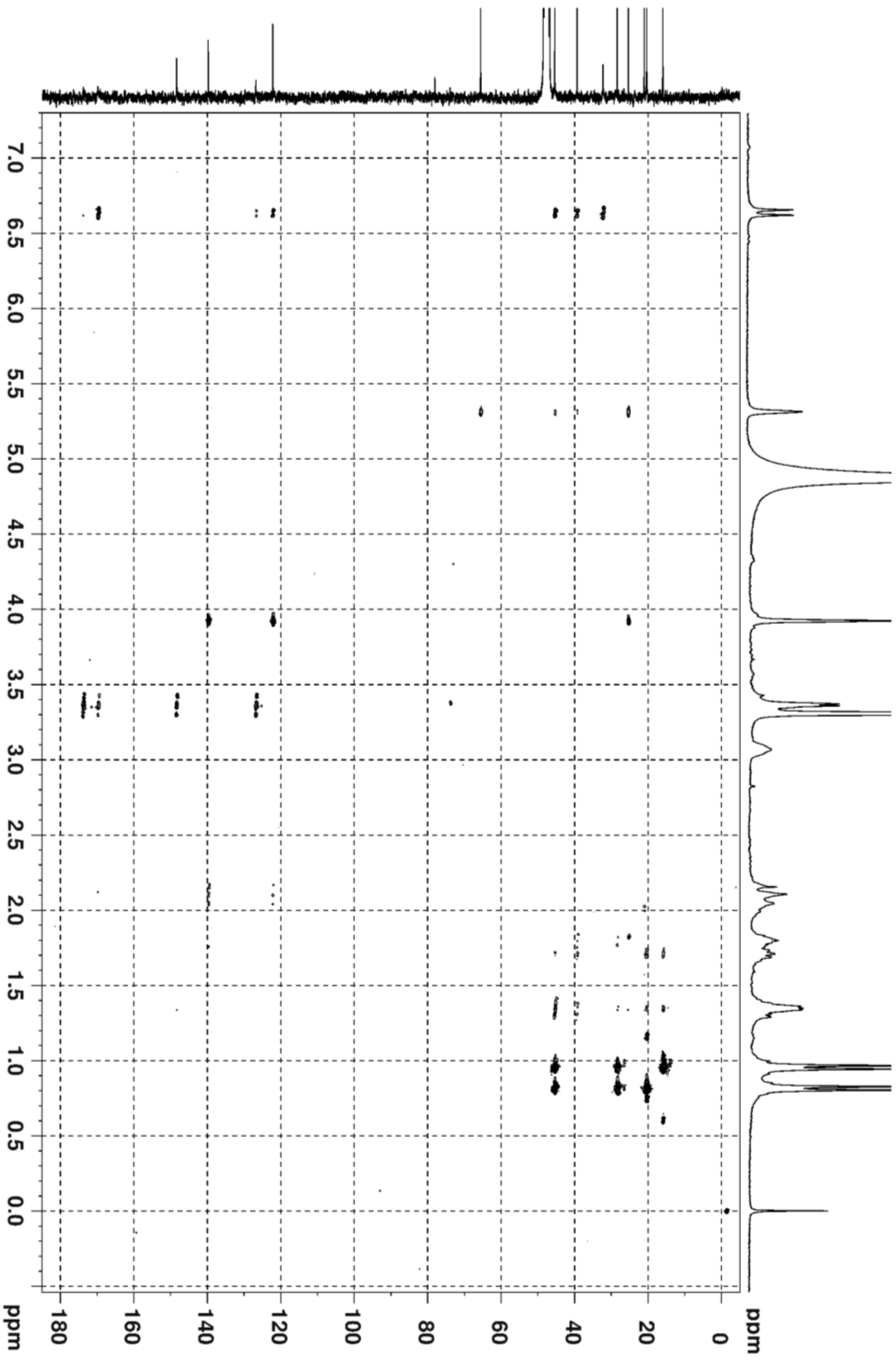

Figure S4. HMBC spectrum (CD_3OD) of compound **1**

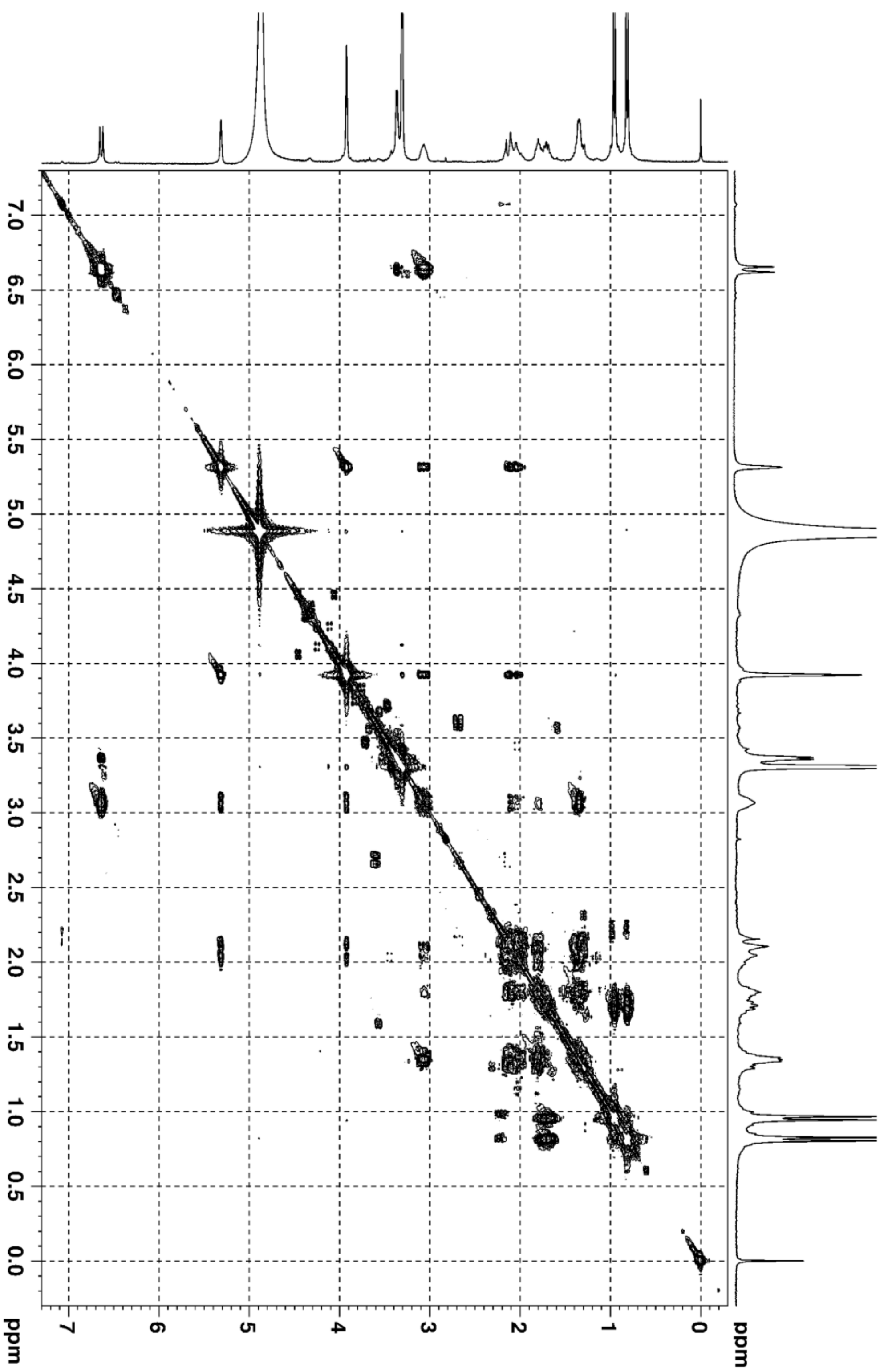

Figure S5. ^{1}H-^{1}H COSY spectrum (CD_3OD) of compound **1**

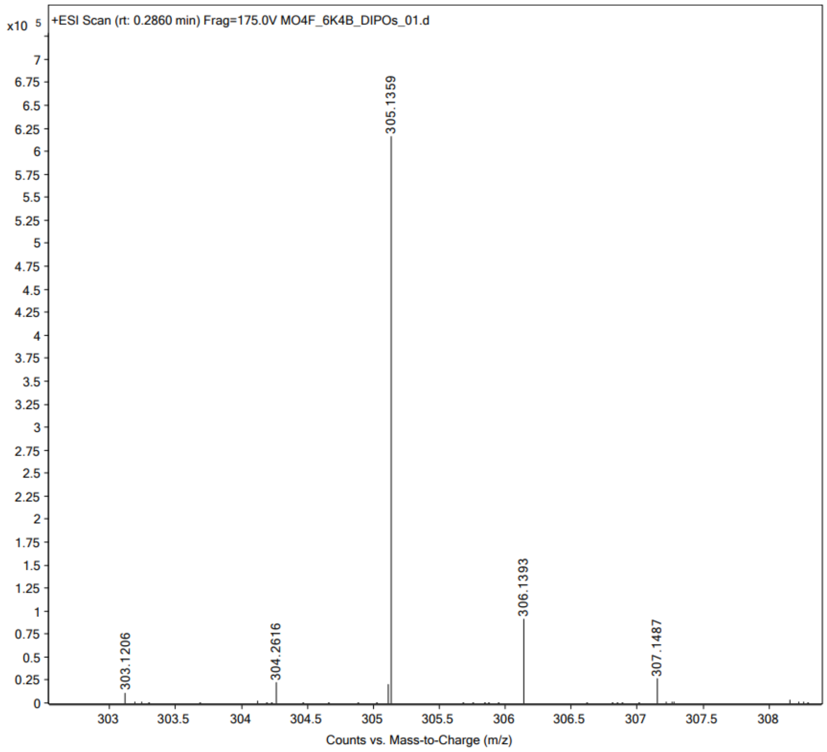

Figure S6. HRESI mass spectrum of compound **1**

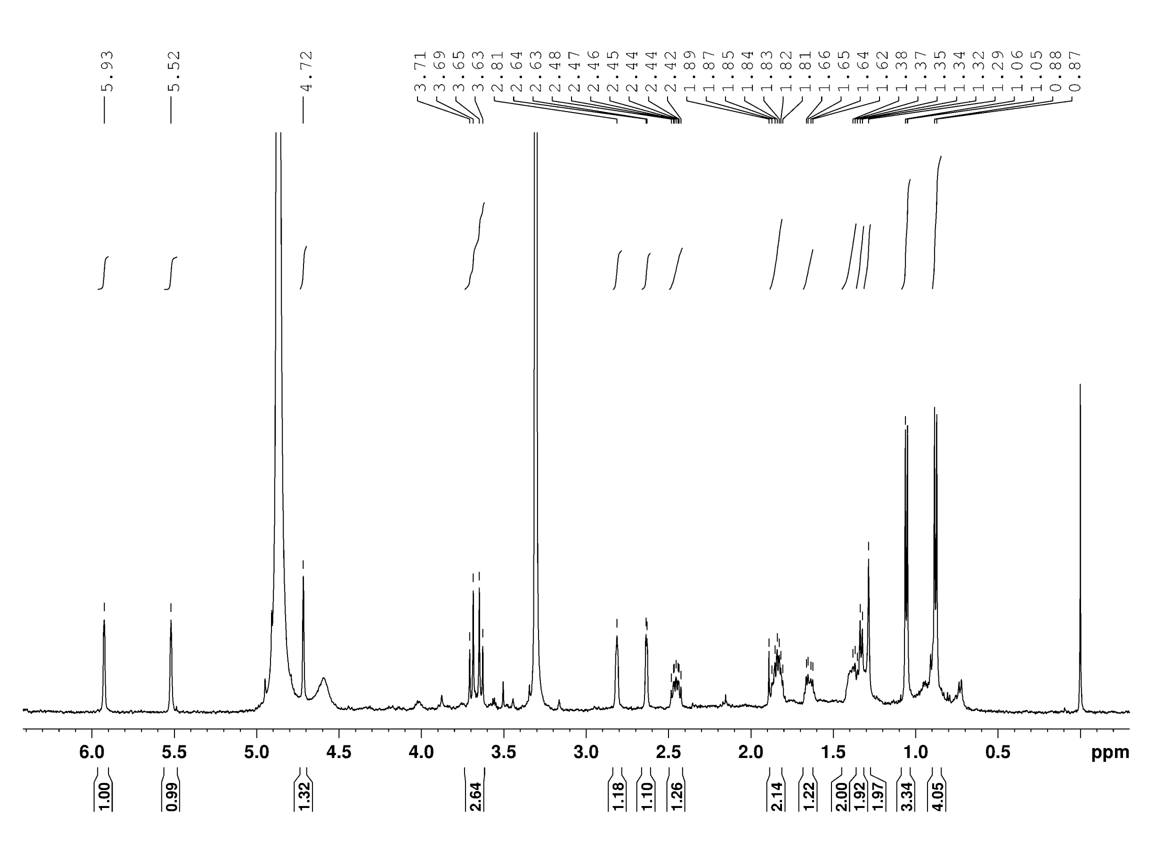

Figure S7. ^{1}H NMR spectrum (500 MHz, CD_3OD) of compound **2**

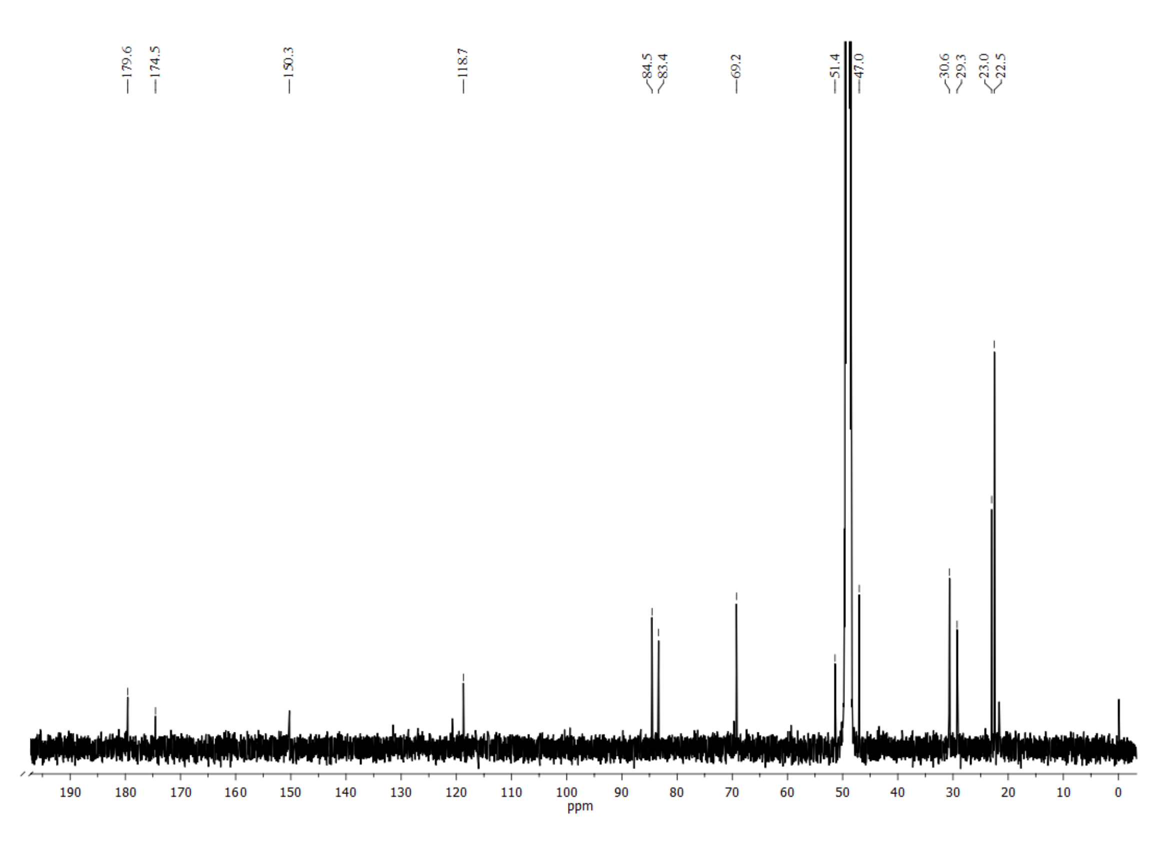

Figure S8. ^{13}C NMR spectrum (125 MHz, CD_3OD) of compound **2**

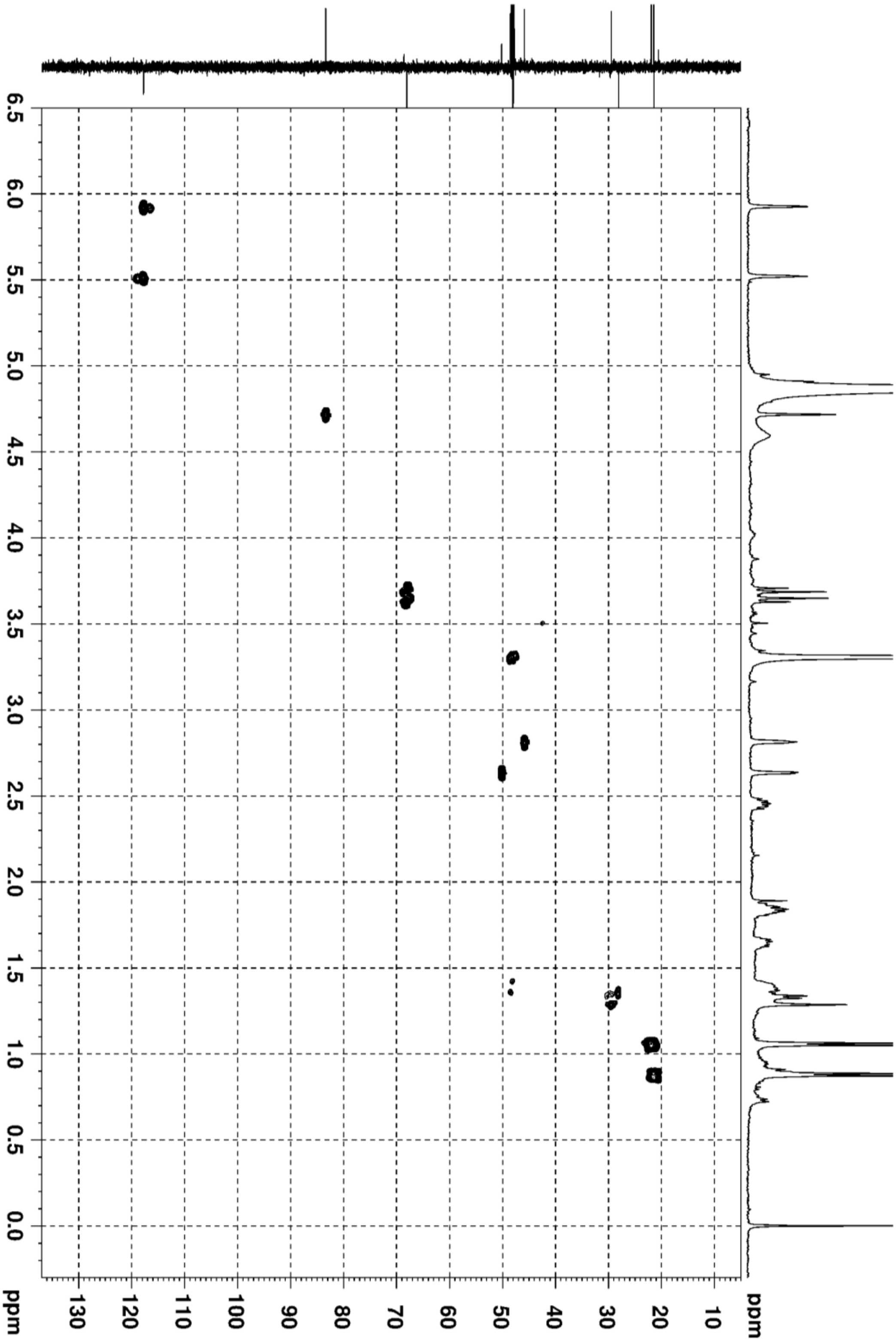

Figure S9. HMQC spectrum (CD_3OD) of compound **2**

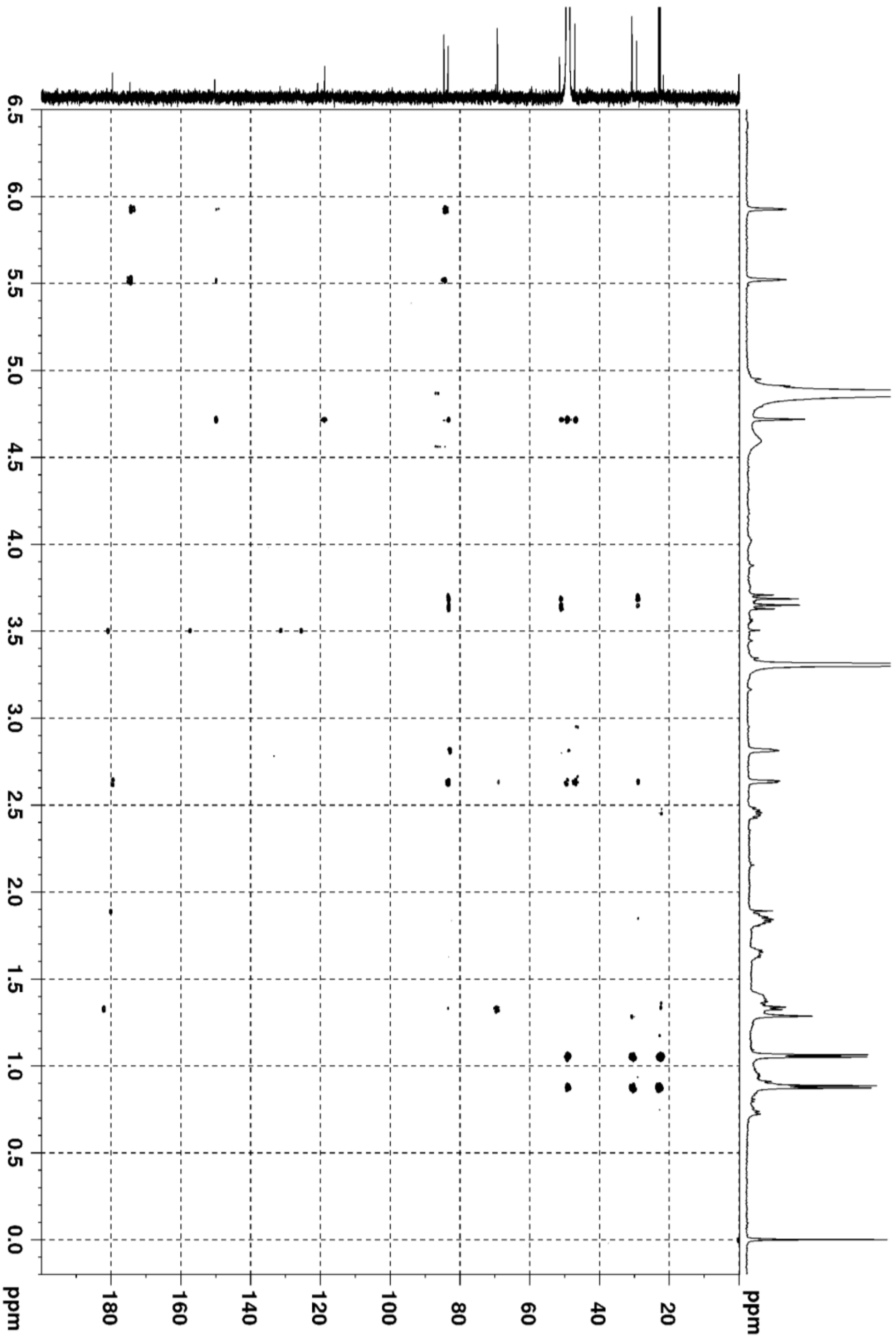

Figure S10. HMBC spectrum (CD_3OD) of compound **2**

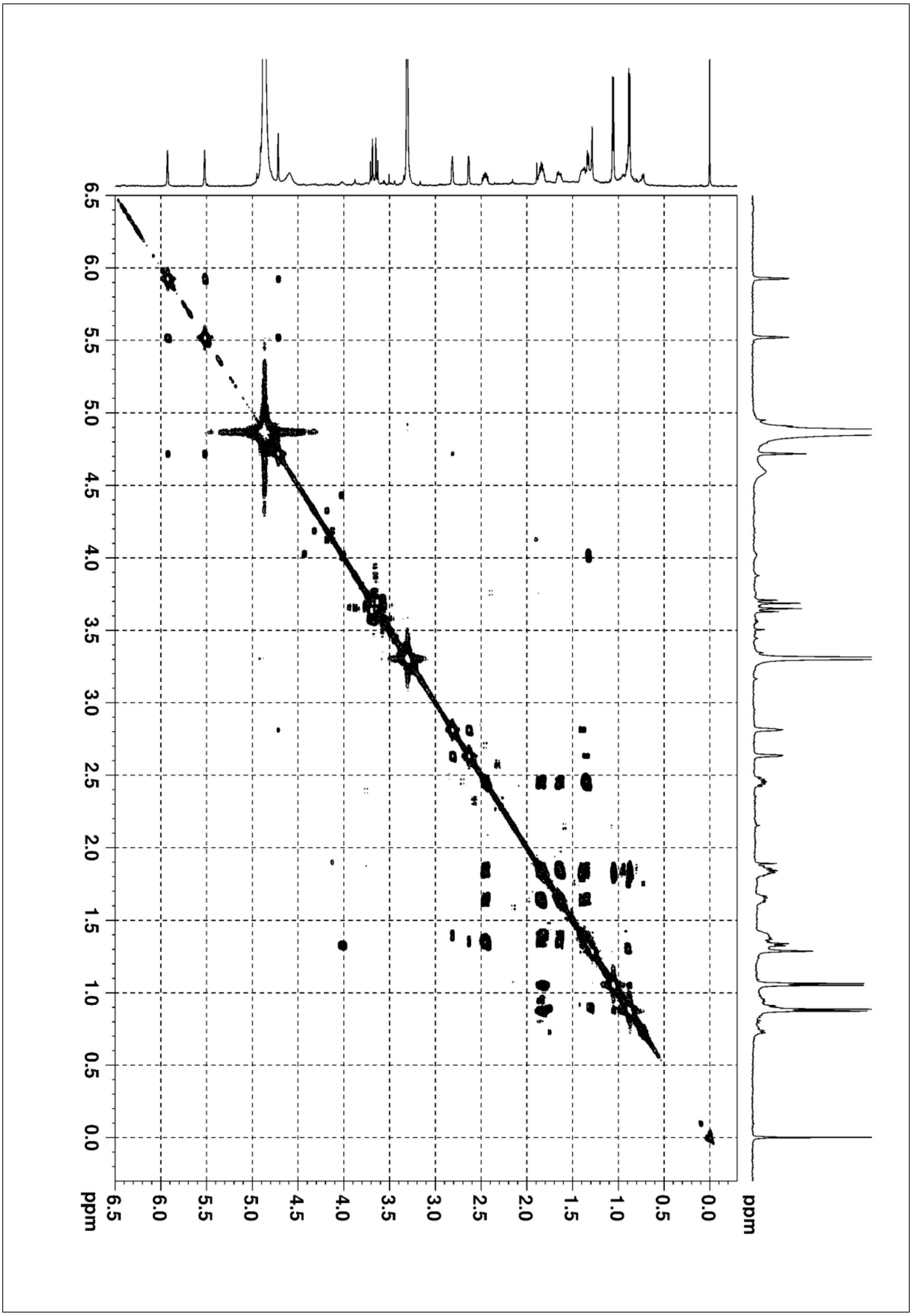

Figure S11. ^{1}H-^{1}H COSY spectrum (CD_3OD) of compound **2**

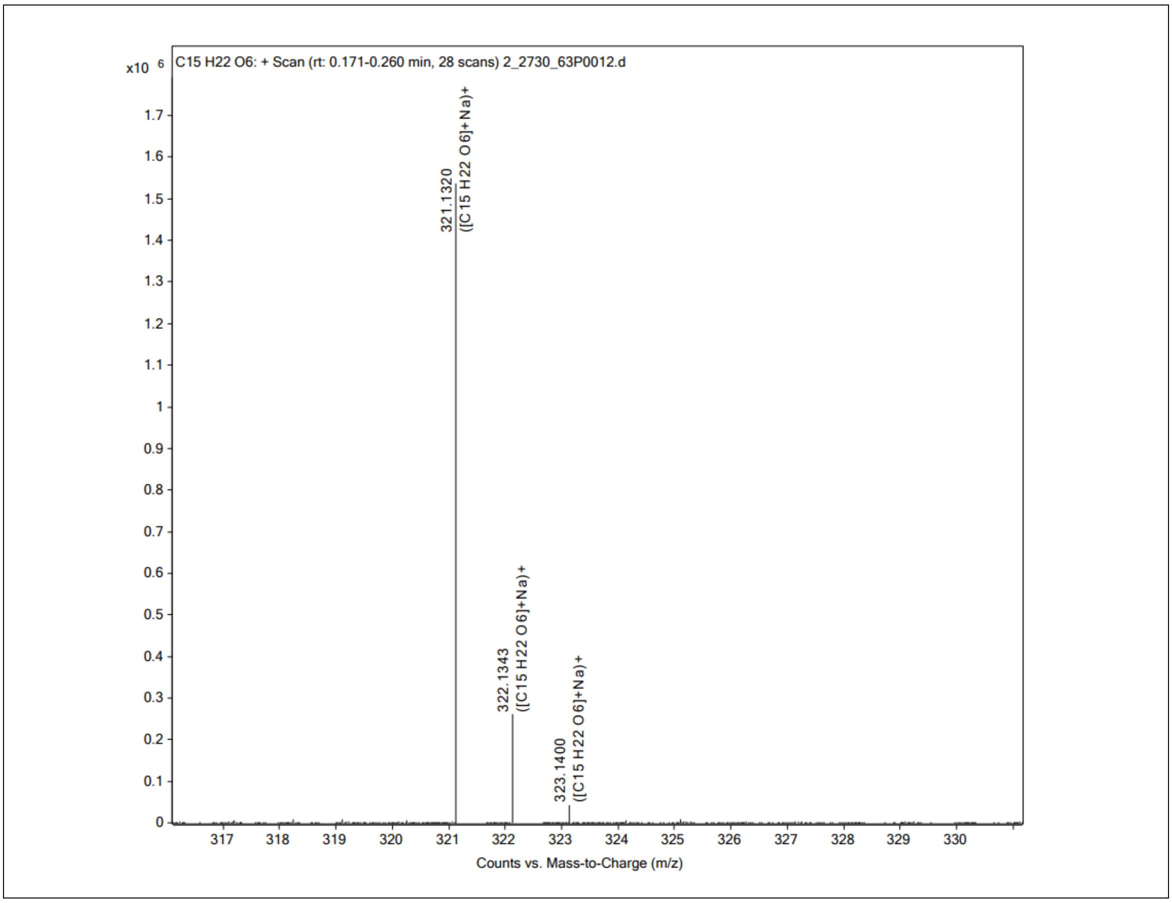

Figure S12. HRESI mass spectrum of compound **2**

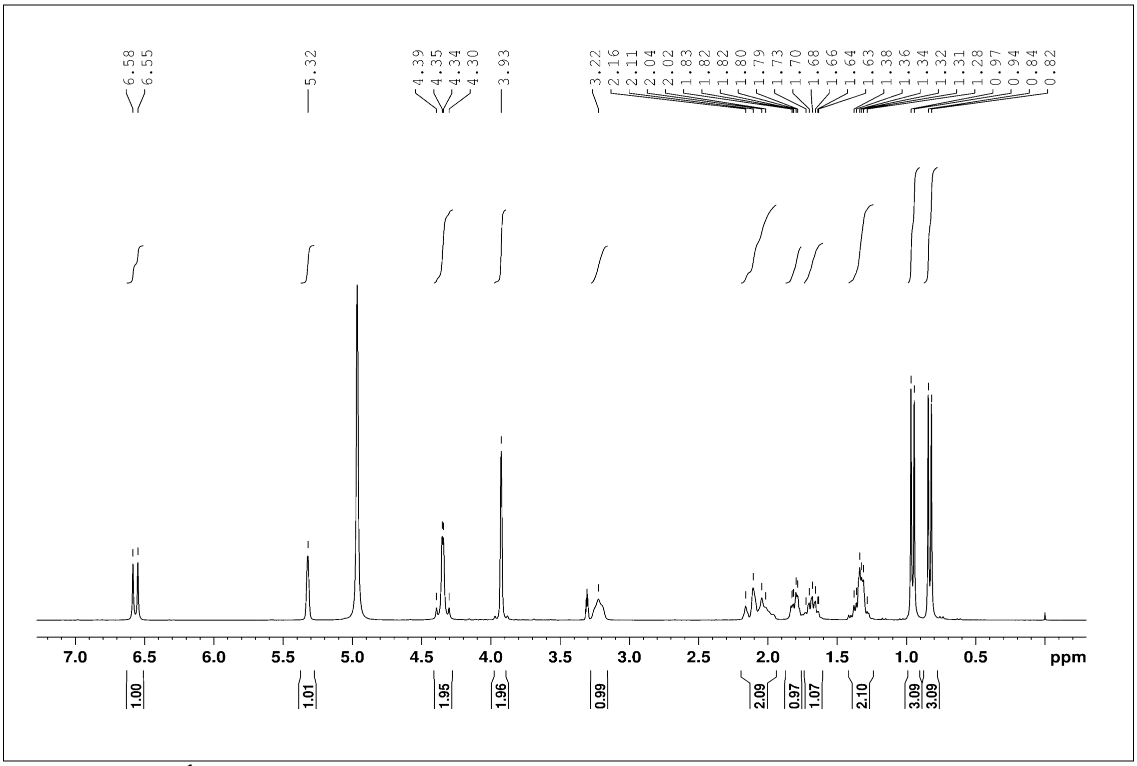

Figure S13. ^{1}H NMR spectrum (300 MHz, CD_3OD) of compound **3**

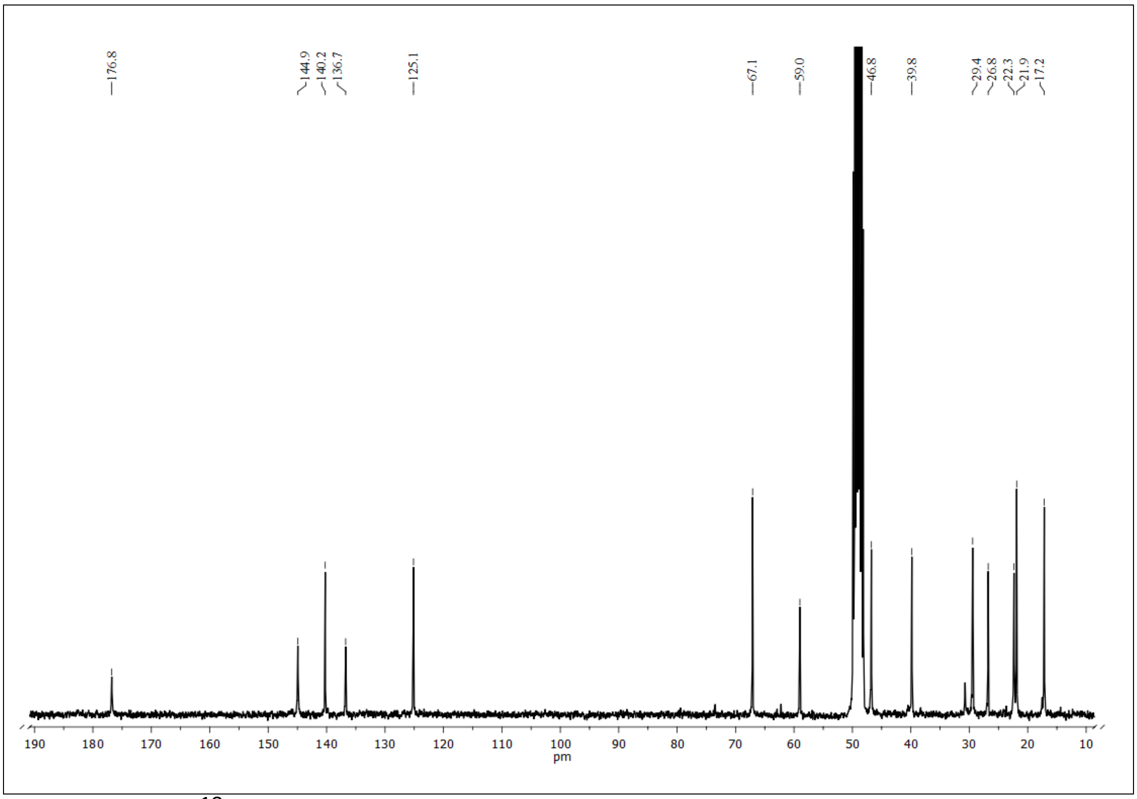

Figure S14. ^{13}C NMR spectrum (75 MHz, CD_3OD) of compound **3**

Colorless gum; $[\alpha]_D^{25}$ +44.3 (*c* 0.67, MeOH), Ref. $[\alpha]_D^{25}$ +41.4 (*c* 0.67, MeOH) [1]; UV (MeOH) λ_{max} (log ε) 210 (3.72); ECD (*c* 0.0008 M, MeOH) λ_{max} (Δε): 227 (+4.6) nm, Ref. ECD (*c* 0.0008 M, MeOH) λ_{max} (Δε): 227 (+4.8) nm [1]; IR (neat) v_{max} 3429, 1656, 1640 cm^{-1}.

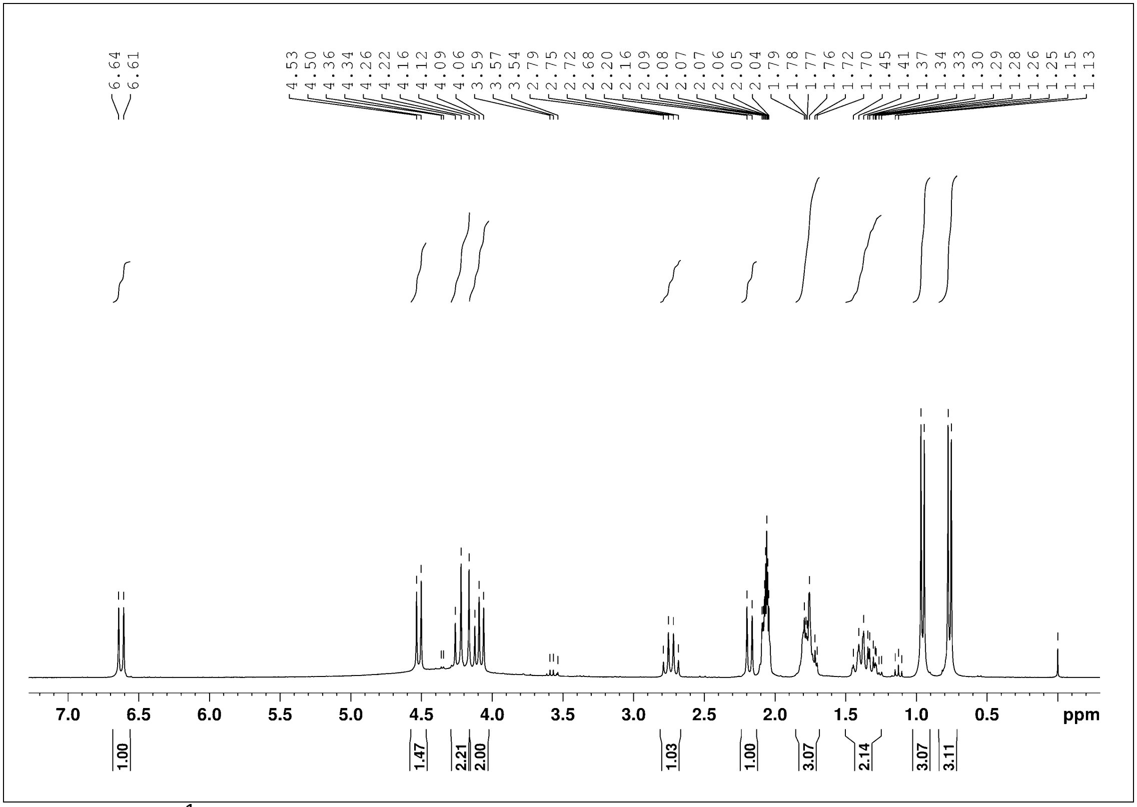

Figure S15. ^{1}H NMR spectrum (300 MHz, acetone-d_6) of compound **4**

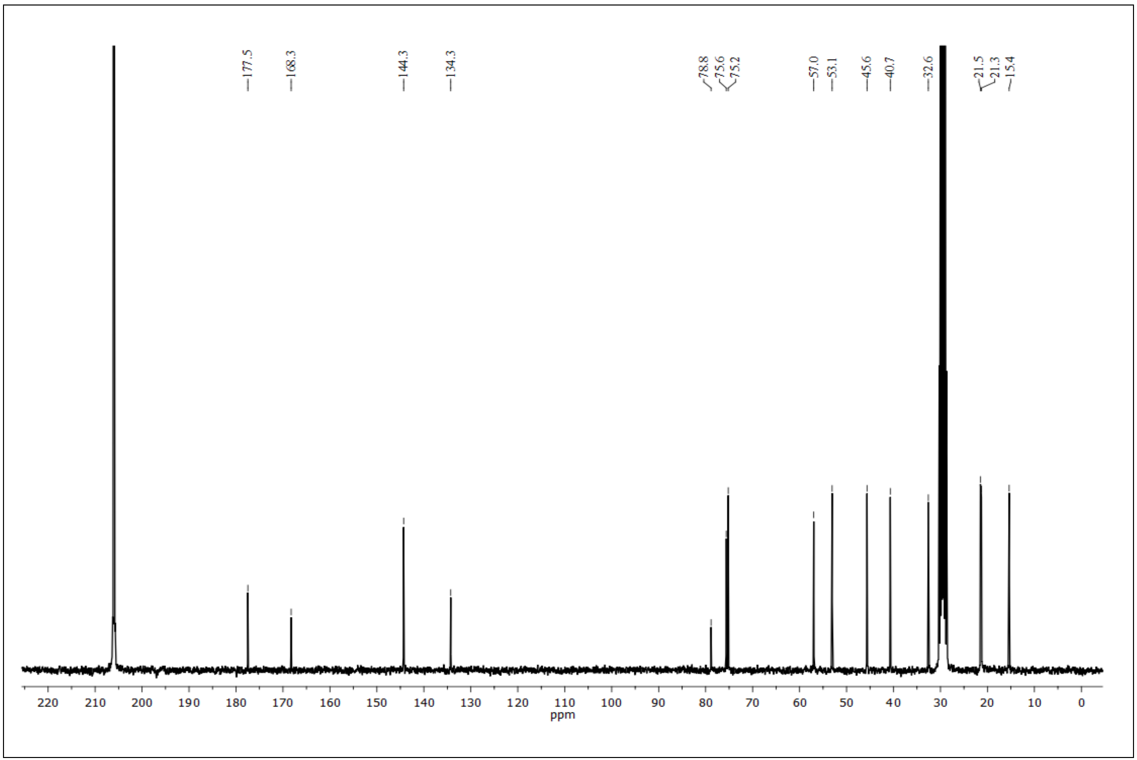

Figure S16. ^{13}C NMR spectrum (75 MHz, acetone-d_6) of compound **4**

Colorless gum; $[\alpha]_D^{25}$ +39.8 (*c* 0.83, MeOH), Ref. $[\alpha]_D^{25}$ +37.0 (*c* 0.83, MeOH) [1]; UV (MeOH) λ_{max} (log ε) 210 (4.14); ECD (*c* 0.0007 M, MeOH) λ_{max} (Δε): 209 (+11.7), 230 (−5.0) nm, Ref. ECD (*c* 0.0007 M, MeOH) λ_{max} (Δε): 208 (+9.1), 230 (−4.8) nm [1]; IR (neat) ν_{max} 3407, 1761, 1684, 1654 cm^{-1}.

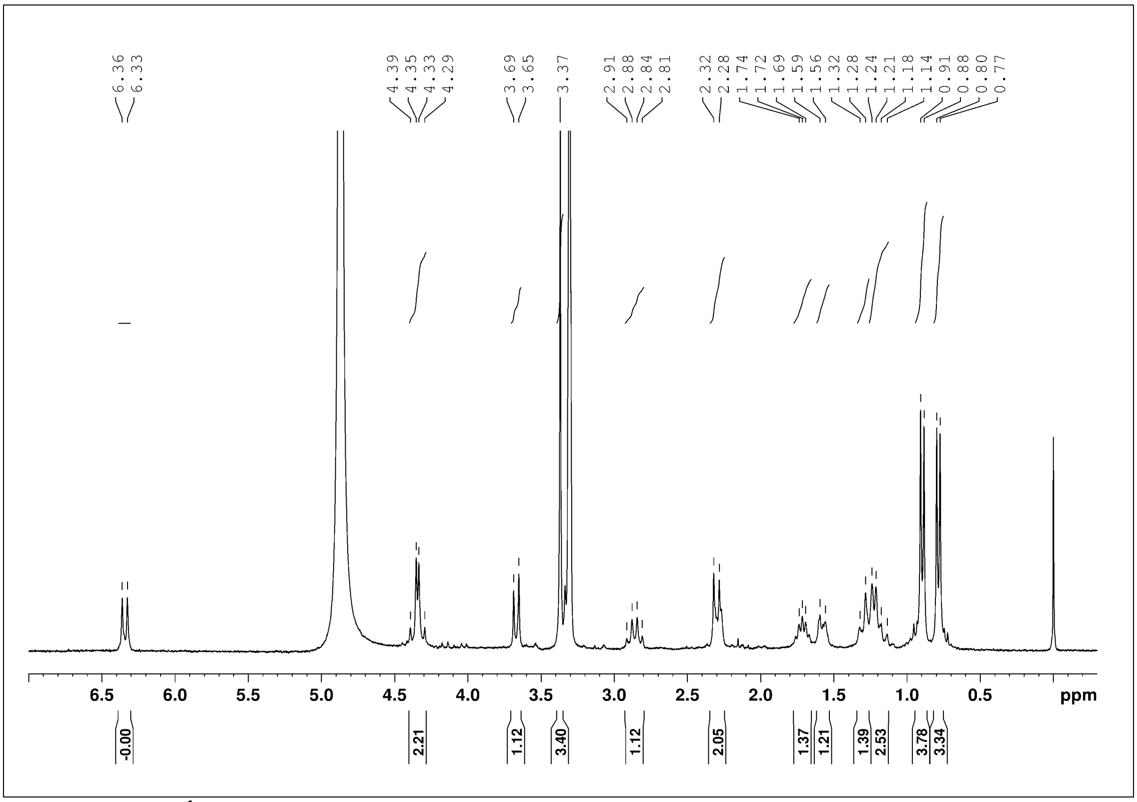

Figure S17. ^{1}H NMR spectrum (300 MHz, CD_3OD) of compound **5**

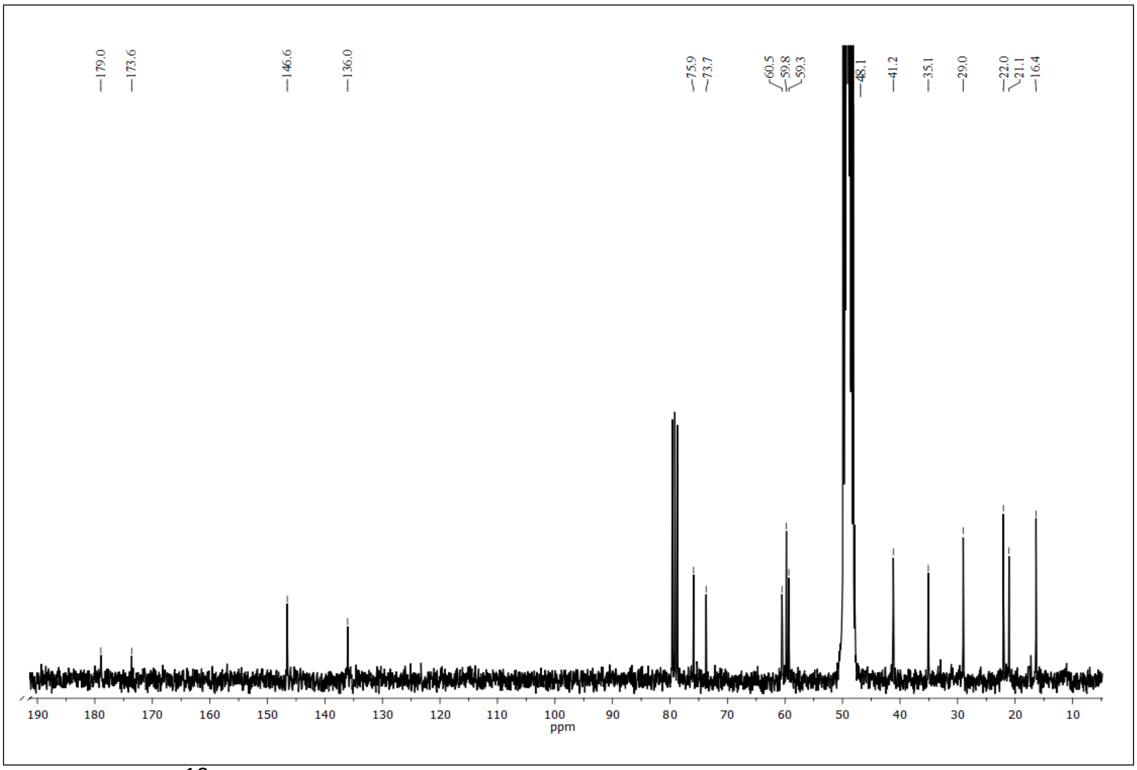

Figure S18. ^{13}C NMR spectrum (75 MHz, CD_3OD) of compound **5**

Colorless gum; $[\alpha]_D^{25}$+30.2 (*c* 0.79, MeOH), Ref. $[\alpha]_D^{25}$+28.0 (*c* 0.79, MeOH) [1]; UV (MeOH) λ_{max} (log ε) 210 (4.14); ECD (*c* 0.0006 M, MeOH) λ_{max} (Δε): 214 (+7.3), 235 (−5.5) nm, Ref. ECD (*c* 0.0006 M, MeOH) λ_{max} (Δ*ε*): 209 (+1.8), 244 (−1.2), 270 (1.0), 289 (−1.1) nm [1]; IR (neat) ν_{max} 3422, 1686, 1639 cm^{-1}.

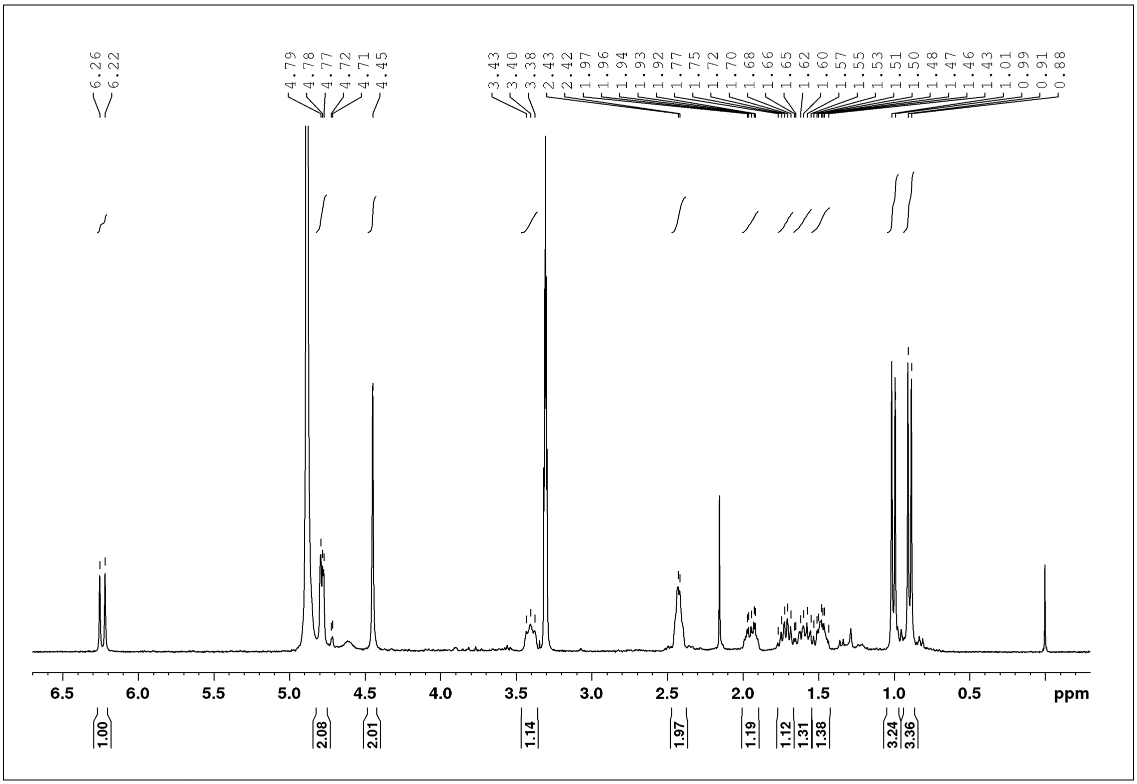

Figure S19. ^{1}H NMR spectrum (300 MHz, CD3OD) of compound **6**

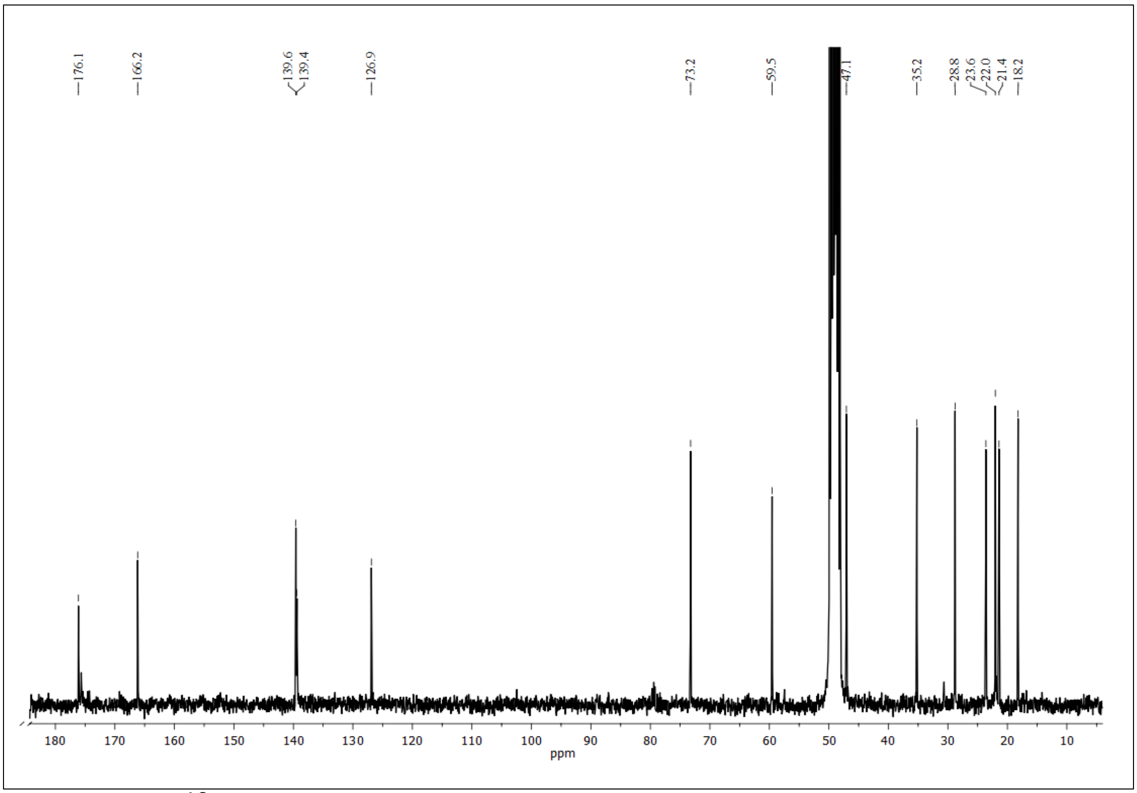

Figure S20. ^{13}C NMR spectrum (75 MHz, CD3OD) of compound **6**

Colorless gum; $[\alpha]_D^{25}$ +75.6 (*c* 0.70, MeOH), Ref. $[\alpha]_D^{25}$ +74.4 (*c* 0.70, MeOH) [1]; UV (MeOH) λ_{max} (log ε) 219 (4.26); ECD (*c* 0.0007 M, MeOH) λ_{max} (Δε): 225 (+11.6), 248 (−1.4) nm, Ref. ECD (*c* 0.0007 M, MeOH) λ_{max} (Δε): 225 (+22.9), 248 (−3.4) nm [1]; IR (neat) ν_{max} 3423, 1684, 1638 cm^{-1}.

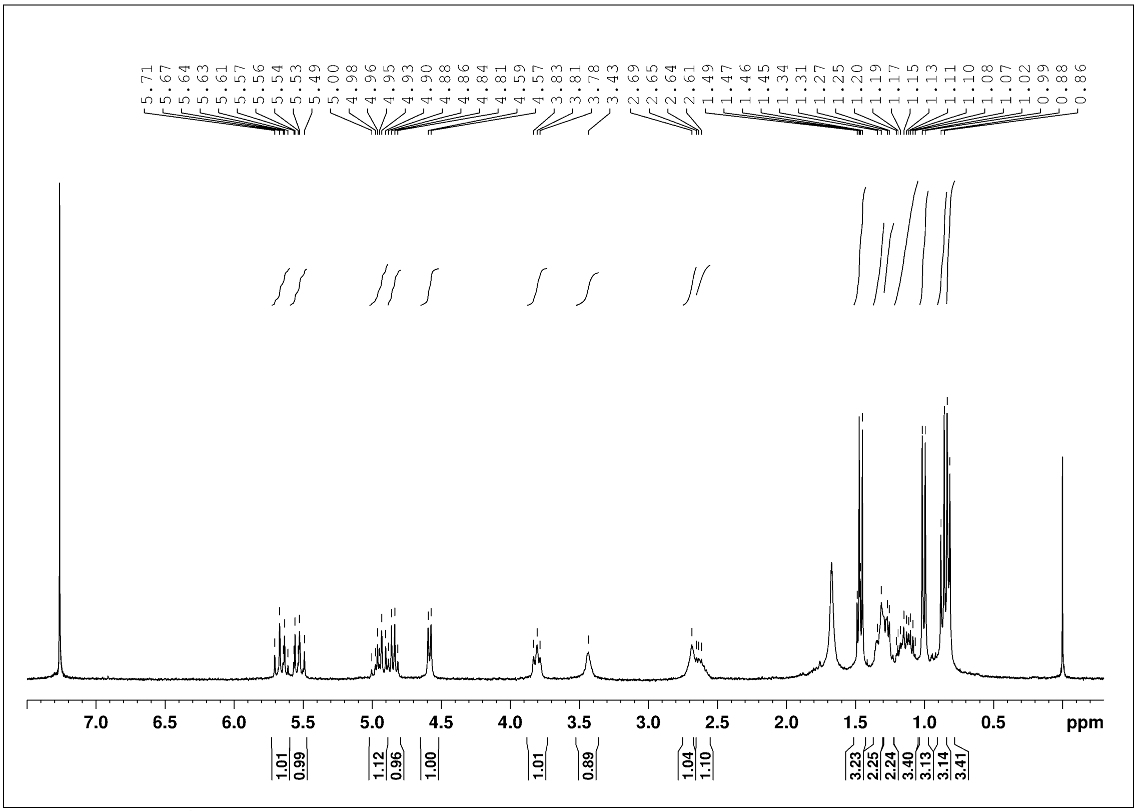

Figure S21. ^{1}H NMR spectrum (500 MHz, $CDCl_3$) of compound **7**

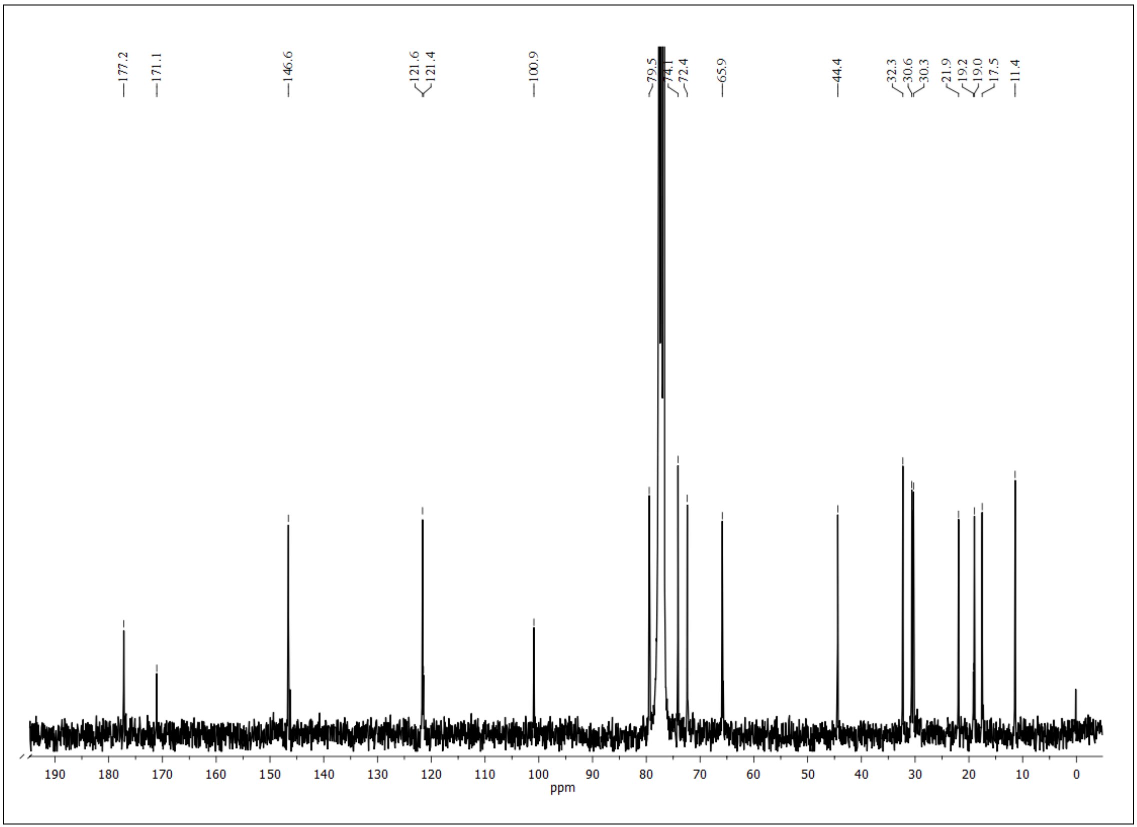

Figure S22. ^{13}C NMR spectrum (125 MHz, $CDCl_3$) of compound **7**

Colorless gum; $[\alpha]_D^{25}$+9.5 (*c* 1.2, CH_2Cl_2), Ref. $[\alpha]_D^{25}$+8.2 (*c* 1.2, CH_2Cl_2) [2]; UV (MeOH) λ_{max} (log ε) 228 (4.15); IR (neat) ν_{max} 3423, 1684, 1638 cm^{-1}.

References

1. Yan, S.; Li, S.; Wu, W.; Zhao, F.; Bao, L.; Ding, R.; Gao, H.; Wen, H.-A.; Song, F.; Liu, H.-W. *Chem. Biodiversity* **2011,** *8*, 1689–1700. doi:10.1002/cbdv.201100026
2. Krohn, K.; Biele, C.; Drogies, K.-H.; Steingröver, K.; Aust, H.-J.; Draeger, S.; Schulz, B. *J. Org. Chem.* **2002,** *14*, 2331–2336. doi:10.1002/1099-0690(200207)2002:14<2331::AID-EJOC2331>3.0.CO;2-P
